# The Outcome of Treatment in Second Branchial Cleft Anomalies: A Case Series

**DOI:** 10.7759/cureus.40164

**Published:** 2023-06-09

**Authors:** Indranil Paul, S.M. Azeem Mohiyuddin, Sagayaraj A, Kouser Mohammadi, Prashanth Babu

**Affiliations:** 1 Otorhinolaryngology & Head and Neck Surgery, Sri Devaraj Urs Academy of Higher Education and Research, Kolar, IND

**Keywords:** surgical complications, recurrence, neck crease incision, branchial fistula, branchial sinus, branchial cyst

## Abstract

Background

Branchial-cleft anomalies are second only to thyroglossal duct anomalies among congenital malformations of the neck, and second branchial-cleft anomalies are the most common. These include branchial cysts, branchial sinuses, and branchial fistulas. Clinical symptoms include neck swelling and a discharging sinus or fistula opening. In a small number of cases, they can lead to major complications like abscesses or malignant changes. Surgical resection is the treatment of choice. Various approaches to resection and sclerotherapy have been tried. In this study, we present our treatment outcome with branchial cleft anomalies at a rural tertiary medical care hospital.

Objectives

To document the various presentations, clinical features, and outcomes of treatment with second branchial cleft anomalies.

Methods

This retrospective observational study included 16 patients operated on for second branchial-cleft anomalies. A detailed medical history was elicited, and an accurate clinical examination was done. A contrast-enhanced computed tomography (CECT) scan was done in all cases. A few cases required a fistulogram. The cysts, sinuses, or fistulas were resected en bloc by a single neck crease incision. Primary closure was done in all cases. A recurrence or pharyngocutaneous fistula required axial flap reconstruction. The complications and recurrences were documented.

Result

There were six children and 10 adults in our study. Seven cysts, five sinuses, and four fistulas were present, of which four were iatrogenic. In seven patients, imaging could not show the entire tract. There were four fistulas from the oropharynx to a cutaneous opening in the neck. A complete resection was done for all. Two pharyngocutaneous fistulas were treated with a pectoralis major myocutaneous (PMMC) flap. Three patients had wound dehiscence postoperatively. None of the patients had neurological or vascular injuries.

Conclusion

Second branchial cleft anomalies can be completely excised by a single neck crease incision. Meticulous surgery results in a low recurrence or complication rate. Following complete excision, in type IV anomalies, a purse-string suture at the pharyngeal opening ensures good closure and no recurrences.

## Introduction

Branchial-cleft anomalies are the second most common congenital malformations of the neck after thyroglossal duct anomalies. Second branchial-cleft anomalies are the most common among them. These include cysts, sinuses, fistulae, or cartilaginous remnants. One of the commonly accepted theories is that the branchial apparatus does not completely involute during embryogenesis [1-4]. Between the fourth and seventh weeks of gestation, the branchial arches are formed, and their derivates include the ear and the muscles, blood vessels, bones, cartilage, and mucosal lining of the face, neck, and pharynx. Mesenchymal invasion destroys clefts and pouches to create different adult structures. When a cleft or pouch does not completely obliterate, it may communicate with either the mucosa of the upper airway or the skin to form a sinus. When both a cleft and pouch fail to obliterate, the communication between skin and mucosa is called a fistula, and when a cleft remnant forms an epithelial-lined space without communication with the mucosa or the skin, a cyst is formed. Most of them are present in the upper half of the neck, but fistulas can present lower down [5].

It can present as a painless, gradually progressive swelling in the lateral aspect of the neck, a discharging sinus, a fistula opening, or recurrent infections [6]. Fistulas are typically discovered in children or infants when fluid or purulent material drains from an opening at the sternocleidomastoid's anterior edge in the neck's lower one-third. Infection of the cyst or fistula tract can lead to pain, purulent discharge, and the spread of infection along tissue planes in the neck [7]. Computed tomography (CT) or magnetic resonance imaging (MRI) confirms the diagnosis, extent, and location of the anomaly, and its proximity to important neurovascular structures. Surgical resection remains the treatment of choice. However, various approaches to resection and differing outcomes by different institutions have still not standardized the treatment. Complete excision is essential for good outcomes. Incomplete excision poses a high risk of recurrence as a fistula or sinus. The various approaches that can be followed are the cervical approach, pull-through branchial fistulectomy, stepladder procedure, stripping method, or a large hockey stick incision in the neck to completely expose and excise the tract [8]. Our series retrospectively analyzed 16 cases of second branchial cleft anomalies and documented the various presentations, clinical features, and outcomes of treatment following resection with a single neck crease incision. This study aimed to document the various presentations and outcomes of treatment for second branchial cleft anomalies.

The objective of this case series was to document the various clinical presentations and outcomes of treatment with regard to complications of surgery and remnants or recurrences of anomalies.

## Materials and methods

This study was approved by the Institution Ethics Committee (IEC): DMC/KLR/IEC/419/2022-23. This is a retrospective analysis of 16 medical case records of patients operated on for second branchial cleft anomalies at a rural medical college hospital from 2003 to 2022.

**Figure 1:**
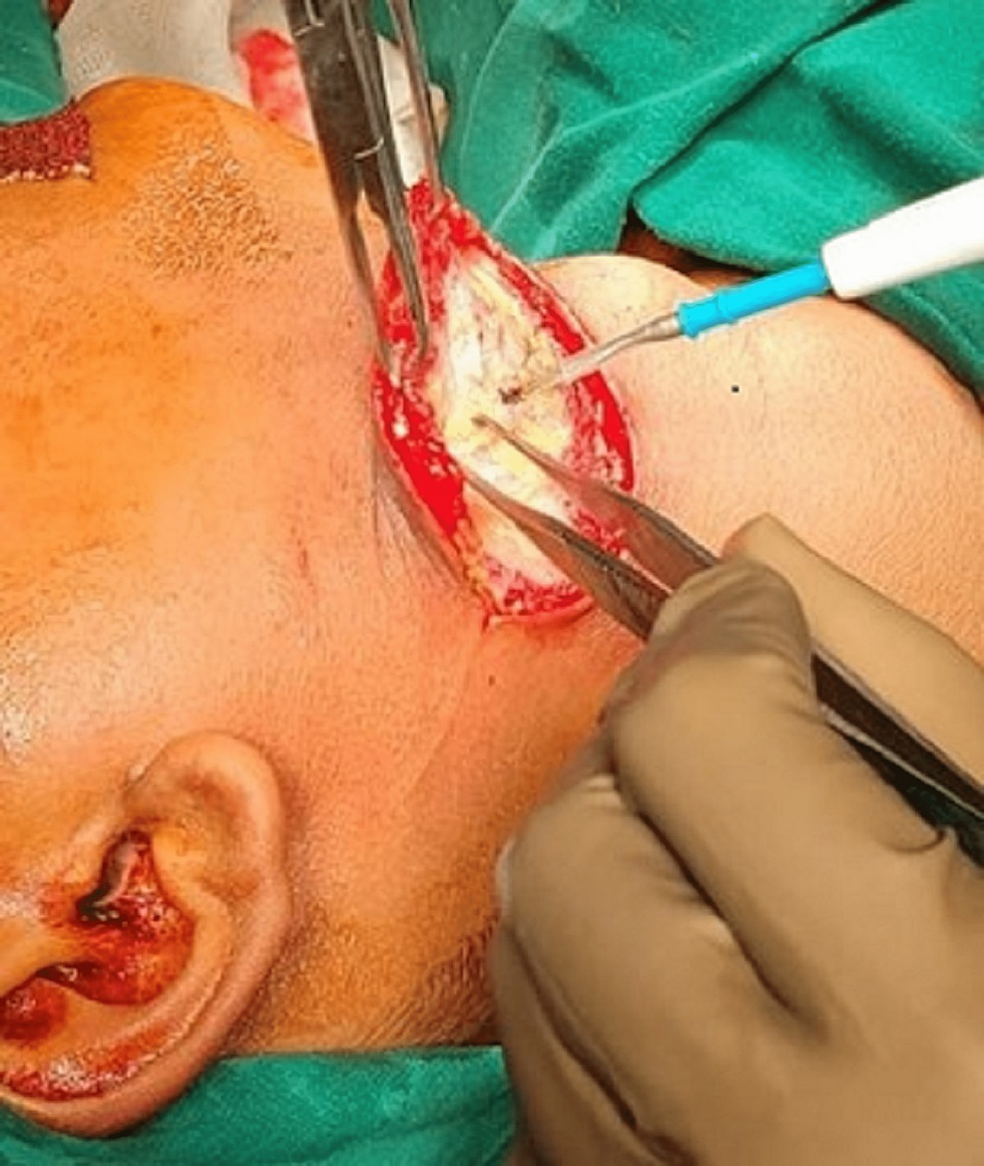
Single neck crease incision over the skin of the neck

Patients diagnosed as having a second branchial cleft anomaly, including branchial cysts, branchial sinuses, and branchial fistulas, were included in this study. Patients with midline neck swelling, thyroglossal tract anomalies, plunging ranula, cystic lymph node, cold abscess, lymphatic cyst, etc., were excluded. A detailed history of the symptoms and earlier treatment was documented, and an accurate clinical examination was performed and the findings documented. A CECT scan was done in all cases. A fistulogram was done in nine patients who had either a sinus or a fistula. The branchial cleft anomalies were resected en bloc by a single neck crease incision in branchial cysts and a similar incision with an ellipse of skin incorporating the external opening in cases of sinuses and fistulas (Figure 1).

**Figure 2:**
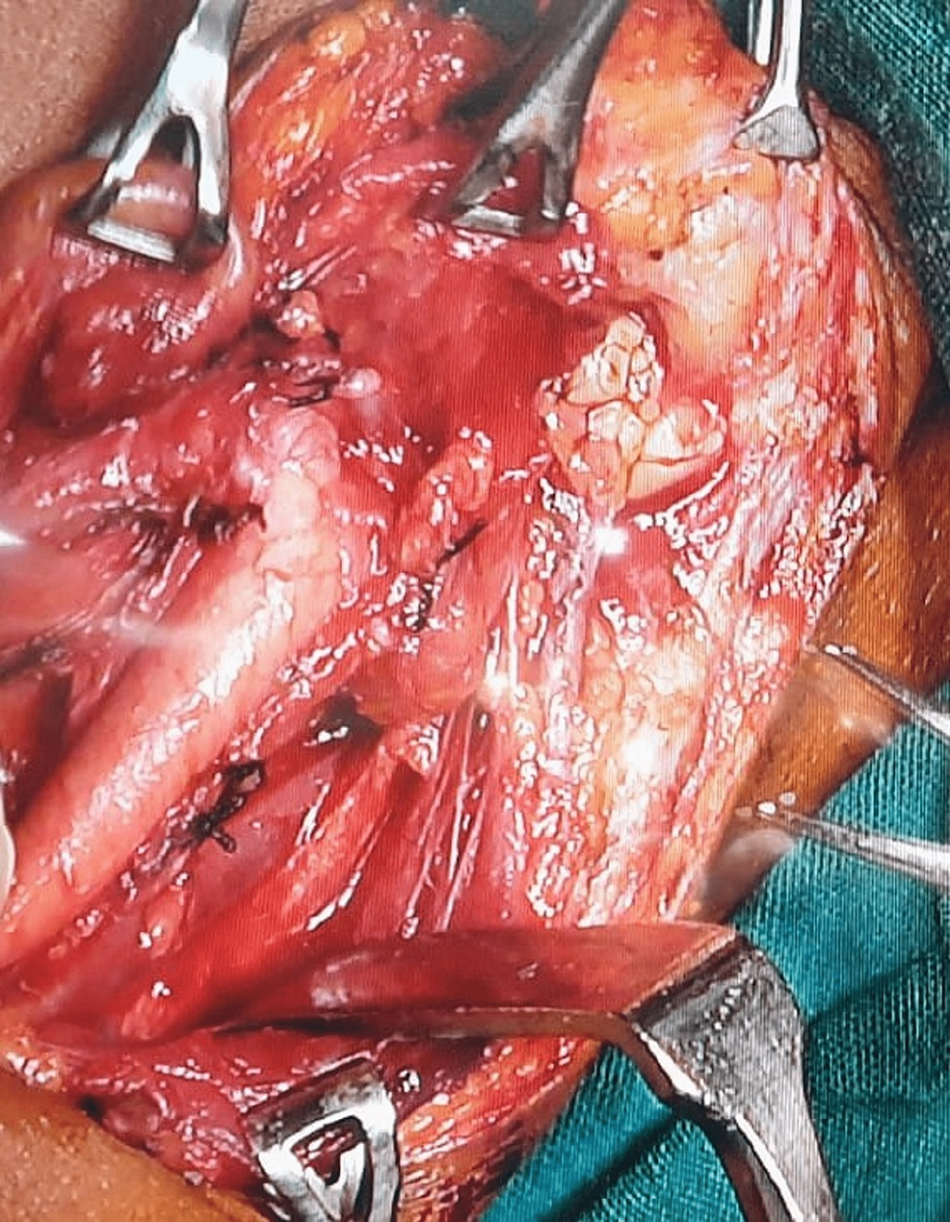
The surgical field after excision of the tract and purse string suture at the oropharyngeal opening.

Methylene blue was injected into the tract to facilitate following the tract during dissection. In cases of fistula, dissection was carried out until the termination of the tract and up to the internal opening in the oropharynx. Internal openings in fistulas were closed using purse string sutures (Figure 2).

Primary closure of the defect and wound was done in most cases, and a PMMC flap was used in two cases as they had wide and infected tracts, and the complications and recurrences were documented. All patients were followed up for a minimum of five years and an average period of eight years. Findings were documented in IBM Corp. Released 2013. IBM SPSS Statistics for Windows, Version 22.0. Armonk, NY: IBM Corp. and descriptive results were analyzed.

## Results

A total of 16 patients underwent surgery for branchial cleft anomalies. They included six children and 10 adults. The age at the time of surgery varied from three years to 55 years, with the mean age being 23.3 years. This case series included eight males and eight females. Fourteen were unilateral (eight on the left and six on the right), and two were bilateral. Two out of the 16 patients had been operated on previously at a different hospital and had presented with recurrence (Table 1).

**Table 1:** Patient demographics, clinical presentation, and outcome. PMMC flap: Pectoralis major myocutaneous flap

Age (years)	Sex	Side	Type of anomaly	Presenting complaints	Surgical Procedure	Post Operative wound dehiscence	Hospital stay
3	Male	left	sinus	painful swelling, discharge	Surgical excision with primary closure	yes	7 days
5	Male	left	cyst	painless swelling	Surgical excision with primary closure	yes	6 days
12	Male	right	cyst	painless swelling	Surgical excision with primary closure	no	6 days
16	Female	left	fistula	painless swelling	Surgical excision with primary closure	no	5 days
17	Female	left	sinus	painful swelling, discharge	Surgical excision with primary closure	no	7 days
18	Female	left	cyst	painless swelling	Surgical excision with primary closure	no	5 days
19	female	right	cyst	painless swelling	Surgical excision with primary closure	no	6 days
20	Female	right	sinus	painful swelling, discharge	Surgical excision with primary closure	no	7 days
20	Male	left	cyst	painless swelling	Surgical excision with primary closure	no	4 days
21	Male	bilateral	recurrent fistula	painful swelling, discharge	Surgical excision with PMMC flap reconstruction	no	14 days
23	Male	left	fistula	painless swelling	Surgical excision with primary closure	no	5 days
30	Male	left	cyst	painful swelling, discharge	Surgical excision with primary closure	no	5 days
32	Female	bilateral	recurrent cyst	painless swelling	Surgical excision with primary closure	no	6 days
35	Female	right	fistula	painless swelling	Surgical excision with PMMC flap reconstruction	no	18 days
48	Female	right	sinus	painful swelling, discharge	Surgical excision with primary closure	yes	6 days
55	Male	right	cyst	painless swelling	Surgical excision with primary closure	no	6 days

**Figure 3:**
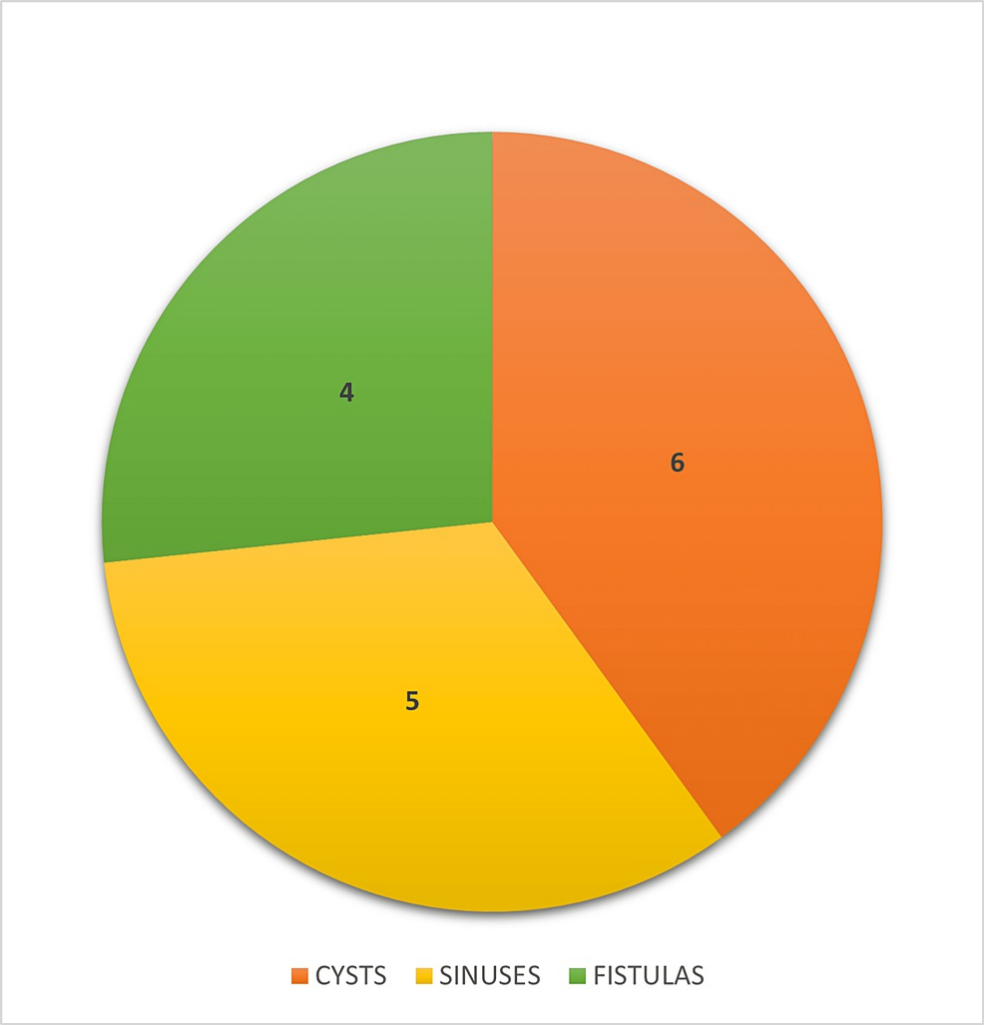
Types of second branchial arch anomalies in our study.

Seven patients, which included five sinuses and two fistulas, did not show the entire tract on imaging. Four had fistulas extending from the oropharynx to a cutaneous opening in the neck. Two cases of infected branchial cysts were given antibiotic prophylaxis before surgery for a minimum duration of one week (Figure 3).

All 16 patients underwent surgical excision through a single neck crease incision. Two of these patients required PMMC flap closure, while the other fourteen had primary closure.

Three patients had wound dehiscence postoperatively, out of whom two were children, including one branchial cyst and one branchial sinus, and one adult with a branchial sinus. All patients received antibiotic prophylaxis post-surgery. The average time taken for wound healing was 10 days; however, it was longer in patients with wound dehiscence. All cases of wound dehiscence healed spontaneously and did not require any further surgical intervention (Table 2).

**Table 2:** Postoperative wound dehiscence.

Patients with wound dehiscence	Type of anomaly	Percentage
Children (n = 6)	1 branchial cyst	16.67%
	1 branchial sinus	16.67%
Adults (n=10)	1 branchial sinus	10%

None of the patients in our series had neurological or vascular injuries. All patients were followed up for a minimum of five years, and none of our patients had recurrences.

## Discussion

Branchial anomalies will most commonly present during childhood but may remain untreated or undetected until later in life due to the non-development of signs and symptoms. They can be a branchial cyst, a branchial sinus, or a branchial fistula [9]. Among the branchial arch anomalies, second branchial arch anomalies are the most commonly encountered ones in clinical practice [4].

In our study, among the 16 cases, seven (44%) were branchial cysts, five (31%) sinuses, and four (25%) fistulas. Studies done by Anoop M. et al. [10] and Zaifullah et al. [11] also show similar findings. The second branchial cleft cysts were divided into four categories by Proctor in 1955. Type I includes superficial cysts that are located beneath the cervical fascia and in front of the sternocleidomastoid muscle. Cysts on the great vessels are classified as Type II (the most frequent); lesions between the internal and external carotid arteries are classified as Type III; and Type IV includes cysts medial to the great vessels and close to the pharyngeal wall [12].

The majority of the cysts and sinuses (eight) were Type II, with a few Type III (four), while fistulas were all Type IV in our study. The two cases in our series that were operated on outside presented with recurrence, which was most likely due to incomplete removal.

The second branchial arch anomaly occurred more commonly on the left side (50%) in comparison to the right side (38%) in our study, with only 12.5% being bilateral. Zaifullah et al. also found that cysts were mostly distributed on the left side in their 10-year retrospective study of branchial cleft anomalies [11].

Susan Muller et al. observed in their description of second branchial cleft cysts that they have no gender predilection [13]. In our series, too, there was no gender predilection, with an equal number of males and females.

The majority of the cases in our study presented in adulthood (62.5%), with the age of presentation ranging between three years and 55 years, with 23.3 years being the mean age at presentation. Susan Miller et al. stated that the majority of the cases presented at the age of 20-40 years, although branchial cleft cysts can present in children less than 5 years of age [13].

Radiologically, a second branchial cleft cyst will present as a round or ovoid mass with an enhancing capsule that is thin, with the content being homogenous fluid [13]. These findings differentiate this benign cystic mass from a metastatic/malignant neck mass, which should be ruled out in all adult patients presenting with a neck mass as the primary complaint. A metastatic cystic lymph node has an irregularly enhancing wall that is thicker in appearance and usually has a central non-enhancing necrotic area. The most crucial differential diagnosis is metastatic cystic squamous cell carcinoma (SCC) to a lymph node from oropharyngeal human papillomavirus (HPV)-associated SCC [13]. However, in our study, none of the patients were found to have malignancy in branchial cysts, and HPV-induced cancers are rare in rural parts of our country.

**Figure 4:**
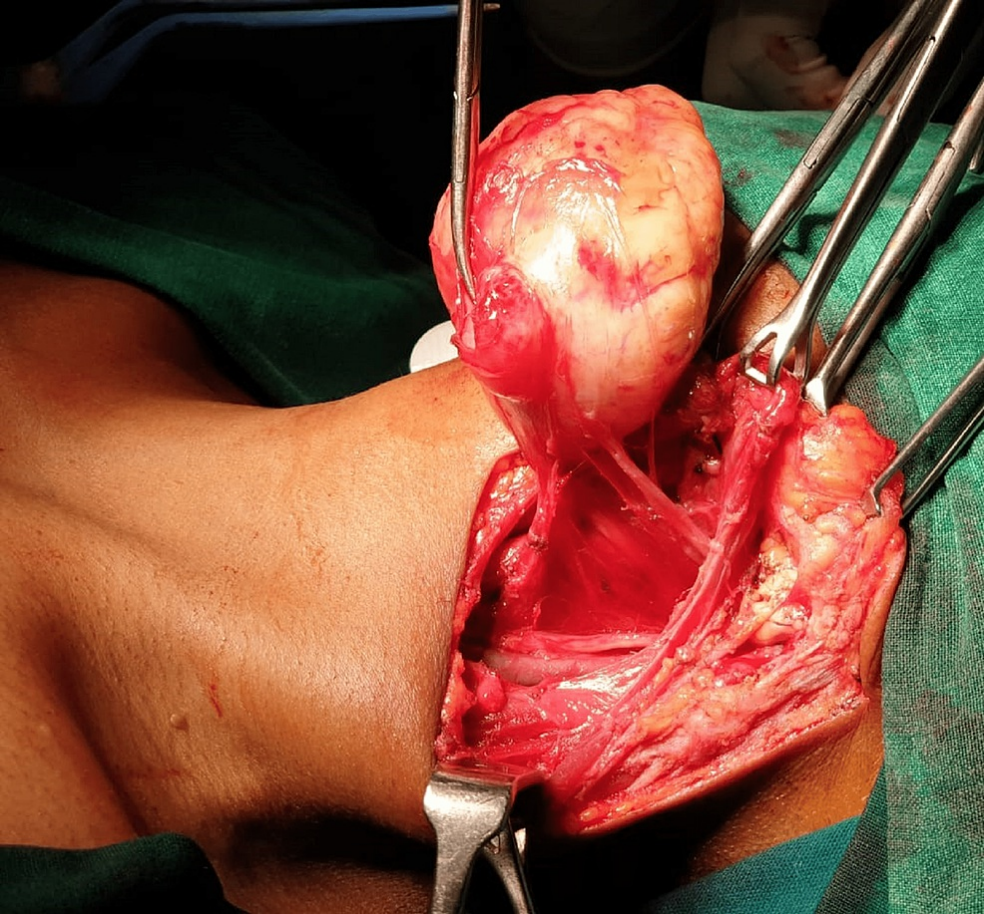
Excision of second branchial cyst.

The mainstay of treatment for a second branchial cleft anomaly is complete surgical excision. The literature review shows that a wide variety of techniques have been followed to get the desired results, such as pull-through branchial fistulectomy, stepladder procedure, stripping method, single large hockey stick incision, and resection by cervical approach. In our series, all patients underwent en-bloc resection by a single neck crease incision and exposure of the anomaly by a subplatysmal flap (Figure 4).

The same procedure as mentioned above, with an ellipse of skin at the external opening, was performed for a fistula or sinus. The tract was followed by injecting methylene blue dye to ensure complete removal. Sclerotherapy was not used at our center as all patients had either a large cyst or a Type III or IV anomaly. Sclerotherapy induces inflammation, and if it fails, surgery post-sclerotherapy has more adhesions, fibrosis, and complications. Ji-Hoon Kim stated in his study that sclerotherapy is more effective for lymphatic malformations than for branchial cleft anomalies. Furthermore, he quoted that sclerotherapy is better for unilocular cysts than multilocular cysts [14]. The sclerosant OK-432 is especially difficult to procure in the Indian subcontinent and more so in a rural setup such as ours [15].

In two cases of our study with Type IV anomalies, the tract was seen to be passing between the carotid bifurcation and crossing between the glossopharyngeal nerve and the hypopharyngeal nerve. The tract was carefully dissected off the carotid artery and hypoglossal nerve, and excision was done en bloc.

In patients who had Type IV anomalies, purse-string sutures following resection at the pharyngeal opening ensured complete removal and could have been the reason that our cases did not have any recurrence. A case series by Wan-Xin Li et al. used purse string sutures in the final step of ligation wherever there was an opening of the branchial fistula into the oropharynx or hypopharynx to prevent infection and recurrence [16].

Some studies suggest unilateral tonsillectomy for complete removal; however, recent studies have disputed this and suggested high ligation of the fistula was sufficient to avoid recurrence. In our study, we did not perform an ipsilateral tonsillectomy in any of our cases [5].

Post-operative complications include injuries to the vagus and recurrent laryngeal nerves, particularly in type III and type IV anomalies, damage to the internal jugular vein, the development of persistent fistulas, local infections, and seroma or hematoma formation [9]. In a study done by Bajaj et al. [17], 3.2% of the patients with second branchial cleft abnormalities experienced post-surgical problems. For the primary excision of branchial abnormalities in children, the literature reports a relapse rate of 3%; however, this rate may increase to 14%-22% for surgical excision following prior infection or prior incomplete excision. [17]. Thus, if an acute infection is present, it is essential to delay surgery and treat it with antibiotics [9]. None of our patients had any major complications or recurrences, though some patients had recently infected anomalies of the second branchial cleft. All our patients underwent surgical excision by open approach. The duration of the hospital stay was less than one week, except for those requiring PMMC flap reconstruction.

Three patients had wound dehiscence, among whom two were children, which could be attributed to their restless nature, and one was a patient with diabetes with high blood glucose levels. None of the patients had any neurological or vascular complications as a result of the wide exposure attained by the approach, the surgery performed by a senior surgeon, and the excellent exposure of the tract by a neck crease incision with subplatysmal flaps. A systematic literature review by Sebastiaan Meijers et al. discusses the complications encountered while using an endoscopic or retro auricular hairline approach, such as temporary earlobe hypoesthesia (7.7%-23.1%) due to perioperative greater auricular nerve manipulation and difficulty of sideward arm raises (27.3%) due to spinal accessory nerve manipulation. On the contrary, patients who underwent the cervical excision technique had a lesser rate of pain and difficulty with sideward arm raises (4.5%) [18]. In a meta-analysis by Che-Fang Ho et al., the duration of surgery was seen to be shorter using the cervical excision technique [19].

Two cases out of four fistulas (type IV anomalies) required pectoralis major myo-cutaneous (PMMC) flap closure for pharyngocutaneous fistula after tract excision. Recurrences of fistulas can only occur when they are not completely excised. Recurrence can occur even many years after surgery. In our study, there was no recurrence in any of the patients after a minimum follow-up period of five years.

Limitations

Our study had a small sample size and was a single-center study.

## Conclusions

Second branchial cleft anomalies can be completely excised by a single neck crease incision, which gives excellent exposure and aids in delineating the entire tract or fistula. The step ladder incision, though small, requires multiple incisions and the stripping method can lead to neurological or vascular injuries. The proximity of important structures in large second branchial cleft anomalies has been associated with complications like injury to the vagus nerve, which was not there in our study as it was meticulously dissected by a senior surgeon, and adequate exposure was achieved by the neck incision. There was no difference in prognosis between children and adults, and the recurrence and complication rates are very low when the anomalies are excised by this approach. Following complete excision, in type IV anomalies, a purse-string suture at the pharyngeal opening ensures good closure and no recurrences.
